# Oral administration of repurposed drug targeting Cyp46A1 increases survival times of prion infected mice

**DOI:** 10.1186/s40478-021-01162-1

**Published:** 2021-04-01

**Authors:** Tahir Ali, Samia Hannaoui, Satish Nemani, Waqas Tahir, Irina Zemlyankina, Pearl Cherry, Su Yeon Shim, Valerie Sim, Hermann M. Schaetzl, Sabine Gilch

**Affiliations:** 1 Calgary Prion Research Unit, Department of Comparative Biology and Experimental Medicine, Faculty of Veterinary Medicine, University of Calgary, 3330 Hospital Drive NW, Calgary, AB T2N 4Z6 Canada; 2 Hotchkiss Brain Institute, Cumming School of Medicine, University of Calgary, Calgary, AB Canada; 3 Department of Medicine - Division of Neurology, Centre for Prions and Protein Folding Diseases, University of Alberta, Edmonton, AB Canada

## Abstract

Prion diseases are fatal, infectious, and incurable neurodegenerative disorders caused by misfolding of the cellular prion protein (PrP^C^) into the infectious isoform (PrP^Sc^). In humans, there are sporadic, genetic and infectious etiologies, with sporadic Creutzfeldt-Jakob disease (sCJD) being the most common form. Currently, no treatment is available for prion diseases. Cellular cholesterol is known to impact prion conversion, which in turn results in an accumulation of cholesterol in prion-infected neurons. The major elimination of brain cholesterol is achieved by the brain specific enzyme, cholesterol 24-hydroxylase (CYP46A1). Cyp46A1 converts cholesterol into 24(S)-hydroxycholesterol, a membrane-permeable molecule that exits the brain. We have demonstrated for the first time that Cyp46A1 levels are reduced in the brains of prion-infected mice at advanced disease stage, in prion-infected neuronal cells and in post-mortem brains of sCJD patients. We have employed the Cyp46A1 activator efavirenz (EFV) for treatment of prion-infected neuronal cells and mice. EFV is an FDA approved anti-HIV medication effectively crossing the blood brain barrier and has been used for decades to chronically treat HIV patients. EFV significantly mitigated PrP^Sc^ propagation in prion-infected cells while preserving physiological PrP^C^ and lipid raft integrity. Notably, oral administration of EFV treatment chronically at very low dosage starting weeks to months after intracerebral prion inoculation of mice significantly prolonged the lifespan of animals. In summary, our results suggest that Cyp46A1 as a novel therapeutic target and that its activation through repurposing the anti-retroviral medication EFV might be valuable treatment approach for prion diseases.

**Supplementary Information:**

The online version contains supplementary material available at 10.1186/s40478-021-01162-1.

## Introduction

Prion diseases, or transmissible spongiform encephalopathies (TSEs), are a group of devastating, infectious and fatal neurodegenerative disorders caused by proteinaceous infectious pathogens termed as prions. Prion diseases affect both humans and animals. The human prion diseases include sporadic Creutzfeldt-Jakob disease (sCJD) as the most common form, genetic forms (e.g. Gerstmann-Sträussler-Scheinker syndrome (GSS) and fatal familial insomnia (FFI)) and acquired forms such as iatrogenic CJD (iCJD) and variant CJD (vCJD). Animal prion diseases include scrapie in sheep, bovine spongiform encephalopathy (BSE) in cattle, and chronic wasting disease (CWD) in cervids (elk, mule deer, white-tailed deer, moose and reindeer) [1–5]. The possibility of zoonotic transmission as demonstrated by BSE and the resulting vCJD, and the unknown potential of CWD to cross the species barrier to humans impose a challenge especially in the absence of therapeutics. Therefore, it is of paramount importance to identify new drug targets and novel therapeutic approaches to treating prion disease.

Prion diseases are caused by the conversion of the cellular prion protein (PrP^C^) into an infectious and misfolded isoform known as PrP^Sc^. PrP^C^ is rich in alpha-helices and sensitive to proteases, while PrP^Sc^ consists mostly of beta-sheets, is prone to aggregate and partially resistant to proteases. Its accumulation in the central nervous system leads to vacuolization and progressive neurodegeneration [6–8]. The mechanism of prion conversion is mostly enigmatic but occurs upon direct interaction of PrP^Sc^ with PrP^C^ which induces the conformational changes. On a subcellular level, prion conversion is thought to take place at the plasma membrane and in the endocytic pathway, mostly in late endosomes/multivesicular bodies and recycling endosomes. Specifically, lipid rafts and the role of cholesterol in prion propagation have been highlighted, and recent work demonstrated that in cultured neurons and in hippocampi of prion-infected mice cholesterol synthesis and the levels of unesterified cholesterol are upregulated [9–14]. The biosynthesis of brain cholesterol takes place in situ and it does not permeate the blood–brain-barrier (BBB) to reach the peripheral circulation [15]. Therefore, aberrant brain cholesterol metabolism is implicated in the pathologies of various protein misfolding neurodegenerative diseases, including prion diseases [12, 16–21]. Prion infection increases cholesterol production resulting in its accumulation in the brain. Furthermore, cholesterol transporter ATP-binding cassette transporter type A1 (ABCA1)-mediated neuronal cholesterol efflux is negatively affected by prion infection, contributing to intracellular accumulation of cholesterol [9–14, 22–25].

The major pathway of brain cholesterol elimination depends on the brain specific enzyme cholesterol 24-hydroxylase (Cytochrome P450 46A1 (Cyp46A1)). The Cyp46A1 converts cholesterol into 24(S)-hydroxycholesterol (24-OHC), a molecule which can permeate the BBB and reach the peripheral circulation system for further metabolism [21, 25–29]. The brain cholesterol homeostasis and turnover (45–50%) is achieved through activation of Cyp46A [30–33]. However, the role of Cyp46A1 in prion diseases has not yet been studied. Therefore, we investigated the expression patterns of Cyp46A1 in brains of mice infected with different prion strains (RML, 22L, and ME7) and in post-mortem brains of sCJD patients. For the first time, we demonstrate that Cyp46A1 protein level is reduced at terminal stages of prion disease in mice independent of the prion strain used for infection, and more importantly, in post-mortem brains of sCJD patients. Hypothesizing that this reduction causes a loss-of-function of Cyp46A1 resulting in increased cholesterol levels upon prion infection, we employed the drug efavirenz (EFV). EFV is a non-nucleoside reverse transcriptase inhibitor that has been used for decades for the chronic treatment of human immunodeficiency virus (HIV) patients. This drug effectively permeates the BBB and has been approved by the food and drug administration (FDA) as an anti-HIV medication since 1998 [34, 35]. Recently, it has been described as an allosteric pharmacological activator of Cyp46A1 [36–41]. Therefore, we argued that EFV treatment will increase the cholesterol export from the brain and reduce prion propagation. We demonstrate here that EFV significantly attenuated PrP^Sc^ propagation in prion-infected neuronal cells and primary neuron cultures and does not affect lipid raft integrity or PrP^C^ lipid raft association. Oral treatment of mice with EFV starting weeks to months after intracerebral prion inoculation significantly increased the survival times of prion-infected animals.

In summary, we show for the first time a reduction of Cyp46A1 levels in prion disease, and its value as an accessible therapeutic target. The fact that EFV is an FDA-approved medication indicates translational potential of our findings.

## Materials and methods

### Materials

EFV was purchased from Toronto Research Chemicals Inc., Ontario, Canada. The PK and Pefabloc (protease inhibitor) were purchased from Roche, Germany. All other reagents/chemicals were purchased from Sigma-Aldrich, USA. The PrP monoclonal antibody (mAb) 4H11 used in this study to detect PrP has been previously described [42]. The antibodies anti-Cyp46A1 (ab82814, Abcam, USA), anti-flotillin-1 (610,821, Mouse BD Transduction Laboratories) and anti-β-actin (Cell signaling) were used. Secondary antibody conjugated with peroxidase, goat anti-mouse HRP, was obtained from Jackson Immuno Research, USA.

### Ethics statement

Female FVB mice (6–8 weeks of age; Charles River Lab) and female C57BL/6 J mice (6–8 weeks of age; Jackson Lab) were used in this study. The mice were kept at 12-h (hr)/12-h light/dark cycle and maintained temperature at 23 °C, in an environment with 60 ± 10% humidity. The mice were allowed to access food and water ad libitum. All animal experiments described in this study were approved by the University of Calgary Health Sciences Animal Care Committee (approved protocols: AC14-0165, Bioassay 1; and AC18-0158, Bioassay) according to the guidelines issued by the Canadian Council for Animal Care and ARRIVE guidelines.

### Animal bioassay I

After acclimatization, FVB mice were randomly divided into four groups of *n* = 4 and intracerebrally (i.c.) inoculated with brain homogenate (1% w/v in PBS; 20 μl) of non-prion infected mice or terminally sick mice infected with RML, 22-L, and ME-7, respectively. The i.c inoculation procedure was performed using 25-gauge disposable needles under anesthesia. Four mice each were euthanized at 75, 100, 125 DPI and at the terminal stage (> 150 DPI) of prion disease. Euthanasia was performed under anesthesia using 5% isoflurane by CO_2_ overdose. Terminal stage was reached when progressive clinical signs of advanced prion disease were observed.

### Animal bioassay II (EFV treatment)

After acclimatization, C57BL/6 J mice were randomly assigned into five groups of *n* = 10 in each group:

1. RML
2. RML + EFV (0 DPI-DW)
3. RML + EFV (30 DPI-DW)
4. RML + EFV (50 DPI-DW)
5. RML + EFV (30 DPI-IP)

All mice were inoculated under anesthesia i.c. into the right parietal lobe with brain homogenate (20 μl of 0.1%) from a RML-inoculated terminally sick mouse using a 25-gauge disposable hypodermic needle. The mice were closely monitored for 10 days for any detrimental effects after i.c. injection. EFV (0.09 mg/kg/day) treatment in drinking water was started on the day of inoculation (0 DPI-DW) or 30 and 50 dpi, according to previously established protocols [19, 41]. For the other post-treatment group, i.p. injection of EFV (0.05 mg/kg/day) was started at 30 DPI 3 times per week for 130 days. After 130 days EFV was delivered at concentration of 0.09 mg/kg/day in drinking water. EFV was dissolved in 0.01% DMSO and added to the drinking water. Every 3 days the EFV solution in drinking water was replaced with fresh EFV solutions and continued until the experimental endpoint. The mice were monitored initially weekly and daily when progressive clinical signs of prion disease were evident. At terminal stage of prion disease, mice were euthanized by CO_2_ overdose under anesthesia and survival times were recorded. Several mice across all groups were euthanized before they reached terminal prion disease for intercurrent diseases and were excluded from statistical analyses (Additional file 1: Table S1).

### Cell lines and EFV treatment

The murine neuroblastoma cell line N2a was obtained from ATCC (CCL-131). Cell cultures were maintained in Opti-MEM Glutamax medium (GIBCO, USA) with 10% fetal bovine serum, and penicillin/streptomycin at 37 °C in a 5% CO_2_ atmosphere. N2a-wt cells are stably transfected cells overexpressing murine PrP. N2a cells persistently infected with mouse-adapted scrapie strains 22L (N2a-22L) or RML (N2a-RML) prions were used in the study [25, 43]. N2a-RML represent a cell line stably overexpressing 3F4-tagged murine PrP [44], in addition to endogenous mouse PrP. CAD5 neuronal cells (a generous gift from Dr. Mahal, Scripps Research Institute, Florida; [45]) were persistently infected with RML or 22L prions (CAD5-RML, CAD5-22L) and cultured at 37 °C in a 5% CO_2_ atmosphere in Opti-MEM Glutamax medium containing 10% bovine growth serum (Hyclone, USA) and penicillin/streptomycin. Cells were treated with EFV at different concentration (5 µM, 10 µM, 20 µM in DMSO) or with vehicle. After 72 h of EFV treatment cell lysates were collected and proceeded for analysis.

### Cerebellar granular neuron (CGN) culture and EFV treatment

Newborn (7 days post-natal) C57BL/6 mice (Charles River, Saint Constant, Quebec, Ca) were used for preparing primary cultures of cerebellar granular neurons (CGN). CGN were mechanically extracted from the cerebella of newborn mice and enzymatically dissociated as previously described [46]. Cells were plated at a density of 1.9 × 10^3^ cells/mm^2^ on plastic culture wells pre-coated with 10 μg/ml poly-D-lysine. Cells were cultured in Dulbecco’s modified Eagle’s medium-Glutamax I high glucose (DMEM) (Life Technologies-Gibco, Ca) supplemented with penicillin and streptomycin (Life Technologies, Ca), 10% fetal bovine serum (Life Technologies, Ca), 20 mM KCl, and N2 and B27 neuronal supplements (Life Technologies, Ca). Cells were incubated at 37 °C in a humidified 5% CO2 atmosphere. Every week, the medium was supplemented with glucose (1 mg/ml) and antimitotics, uridine and fluorodeoxyuridine (10 μM) (Sigma-Aldrich) to reduce astrocyte proliferation in the culture.

After 48 h of seeding, CGNs were infected with RML mouse adapted scrapie prions. As previously described [46], RML-infected mouse brain homogenate (10% w/v in PBS) was sonicated and added at a final concentration of 0.01% to CGN cultures. After four days, the inoculum was removed, cultures were washed twice with media and fresh media was added for the rest of the experiment.

From 1- or 7-day post infection (dpi) as indicated, until the end of the experiment, infected cultures were treated every 4 days with various concentrations of EFV (5 and 10 µM), or with vehicle. Cells were lysed for PrP^Sc^ detection at different days post infection as indicated and lysates were processed for further analysis.

### Cell lysis and proteinase K (PK) digestion

Cell lysis was done as described previously [46, 47]. For PK digestion, lysates were incubated with PK at a final concentration of 20 µg/ml (N2a/CAD5 cells) and 5 µg/ml (CGN) for 30 min at 37 °C. PK digestion was terminated by the addition of Pefabloc protease inhibitor. Proteins were precipitated by adding methanol and resuspended in sample buffer for immunoblot analysis.

### Immunoblot analysis

Immunoblot analysis was performed as previously described [46]. Protein samples were re-suspended in TNE buffer (50 mM Tris–HCl pH 7.5; 150 mM NaCl; 5 mM EDTA) and separated on 12.5% SDS-PAGE. Electroblotting was done using Amersham Hybond P 0.45 PVDF membranes (Amersham, USA). Membranes were incubated with primary and secondary antibodies as indicated and analyzed using Luminata Western Chemiluminescent HRP Substrates (Millipore, USA). The densitometric analysis of immunoblots was performed using ImageJ.

### Preparation of brain homogenates

Brain homogenates (BH) were prepared in PBS (10% w/v) using a gentle MACS™ Dissociator for 2 min at room temperature, followed by centrifugation at 2,000 g for 1 min. The homogenates were aliquoted and stored at − 80 °C until further use. For immunoblotting, 10% BH was mixed with equal volume of cold lysis buffer (10 mM Tris–HCl, pH 7.5, 100 mM NaCl, 10 mM EDTA, 0.5% Triton X-100, 0.5% sodium deoxycholate) and incubated at 4 °C overnight. Resulting 5% BHs were incubated with either water for no PK or 50 µg/ml of PK (final concentration) for digestion at 37 °C for 1 h. PK digestion was stopped by addition of proteinase inhibitors (Pefabloc) to both no PK and PK-digested BHs. This was followed by addition of 3X sample loading buffer and boiling at 95 °C for 5–7 min. The samples were processed for immunoblotting as described above.

### Immunofluorescence staining for Cyp46A1 in mouse brains

Paraffin-embedded mouse brain tissue samples (5 µm-thick sections) on gelatin-coated slides were deparaffinized three times with absolute xylene (5 min for each wash) and rehydrated with graded ethyl alcohol (100% to 70%). Then they were washed twice with TBST (10 mM Tris–HCl (pH 7.4), 150 mM NaCl, 0.05% Tween 20) for 10 min and incubated for 1 h with 2% normal goat serum as a blocking solution and 0.3% Triton X-100 in PBS. After blocking, the slides were incubated with primary antibody (rabbit-Cyp46A1 (ab82814) diluted 1:100 in blocking solution overnight at 4 °C. After primary antibody incubation, the sections were washed twice for 5 min each and incubated for 1 hr with Alexa Fluor 488 goat anti-rabbit or Alexa FluorTM 555 goat anti-rabbit secondary antibodies (Jackson Immunoresearch) (1:100). Coverslips were mounted with Dako fluorescent mounting medium (Molecular Probe, Eugene, OR). The immunofluorescence images were captured at same conditions for all images using a confocal laser scanning microscope (Zeiss LSM 700 confocal microscope). Five images per section (tissue) were captured from each respective group. Confocal images were converted to tagged image file format (TIF). The quantification of the immunofluorescence intensity in the same region of the brain areas (cerebellum, medulla) in the TIF images for all groups was performed using ImageJ software. The background of TIF images was optimized according to the threshold intensity, and the immunofluorescence intensity was analyzed at specified threshold intensity for all groups at same conditions and was expressed as the relative integrated density between the groups.

### Immunofluorescence staining in neuronal cells

The non-infected CAD5 and infected CAD5-RML and CAD5-22L and N2a-RML cells were seeded on coverslips in 12 or 24 well plates. N2a-RML cells were treated with EFV (20 µM) for 3 days for PrP^Sc^ staining. Control cells were treated with DMSO for 3 days. After reaching 70% confluence the cells were fixed with 4% paraformaldehyde in PBS for 20 min at room temperature (RT) and washed three times with PBS. Cells were incubated for 1 h with 10% fetal bovine serum (FBS) as a blocking solution and 0.1% Triton X-100 in PBS. For PrP^Sc^ staining, cells were treated for 7 min with 6 M guanidine hydrochloride, washed 3 times with PBS and further incubated with primary antibody 4H11 or Cyp46A1 diluted 1:100 in blocking solution for 1 h at RT. After primary antibody incubation, cells were washed three times (5 min each) in PBS and further incubated with secondary antibody (Alexa FluorTM 555 goat anti-mouse secondary antibody, Invitrogen -1:500) for 1 h at RT. Nuclei were stained with DAPI for 10 min. Cells were washed with PBS and after final washes, coverslips were mounted on slides using Mounting Medium (PermaFluor™, Thermo fisher). Images were collected and processed using a confocal laser scanning microscope (Zeiss LSM 700 confocal microscope) and all the images were taken using the same conditions. Image analysis and quantification of the mean PrP^Sc^ intensity per nucleus was done using the Zen Desk 3.2 imaging software with the zone of influence (ZOI) method. For Cyp46A1 the number of original confocal images per well of the chamber slide was five per group and the images were converted into TIF images. The fluorescence intensity of the same region of TIF images for all groups was measured using ImageJ software (National Institutes of Health, Bethesda, MD). The TIF image background was optimized according to the threshold intensity and the immunofluorescence intensity at the same threshold intensity for all groups was analysed and was expressed as the relative integrated density of the samples relative to control cells.

### Flotation assays for lipid raft isolation

Lipid rafts were isolated as described previously [11]. Briefly, 3 × 10^7^ N2a-WT cells treated for 3 days with EFV (20 µM) or not were solubilized in 400 μl cold lysis buffer (NaCl 150 mM, Tris–HCl pH 7.5 25 mM, EDTA 5 mM, and Triton-X 100 1%) and incubated on ice in the cold room for 30 min. The cell lysates were mixed with Nycodenz 70% in TNE (NaCl 150 mM, Tris–HCl pH 7.5 25 mM, EDTA 5 mM) to a final concentration of 35% Nycodenz and were overlaid by 200-μl fractions of Nycodenz solutions with concentrations of 25, 22.5, 20, 18, 15, 12, and 8%. After ultracentrifugation (200,000 g, 4 h, 4 °C, Beckmann TLS55 rotor), fractions were collected from the top to the bottom of the gradient and precipitated with methanol for immunoblot analysis.

### Data and statistical analyses

Statistical analyses and histograms were produced using GraphPad Prism software (GraphPad 8, Software, USA). For statistical analysis of immunoblot signals two-tailed independent Student’s t-test for two groups or for multiple groups, one-way analysis of variance (ANOVA) followed by Turkey’s post hoc test or Dunnett’s multiple comparison test, as applicable, was used. Values are expressed as mean ± SEM. The graphical representation of survival times of animals was done using a Kaplan–Meier plot. The log rank test was used for statistical analysis of differences between groups in the survival plot with median and pairwise comparisons between control and treated group. Mice euthanized for reasons not related to prion disease were excluded from the analysis. Significance = *p ≤ 0.05, **p ≤ 0.01, and ***p ≤ 0.001.

## Results

### Reduced Cyp46A1 level at terminal stages of prion disease in prion-infected mice and sCJD patients

**Fig. 1:**
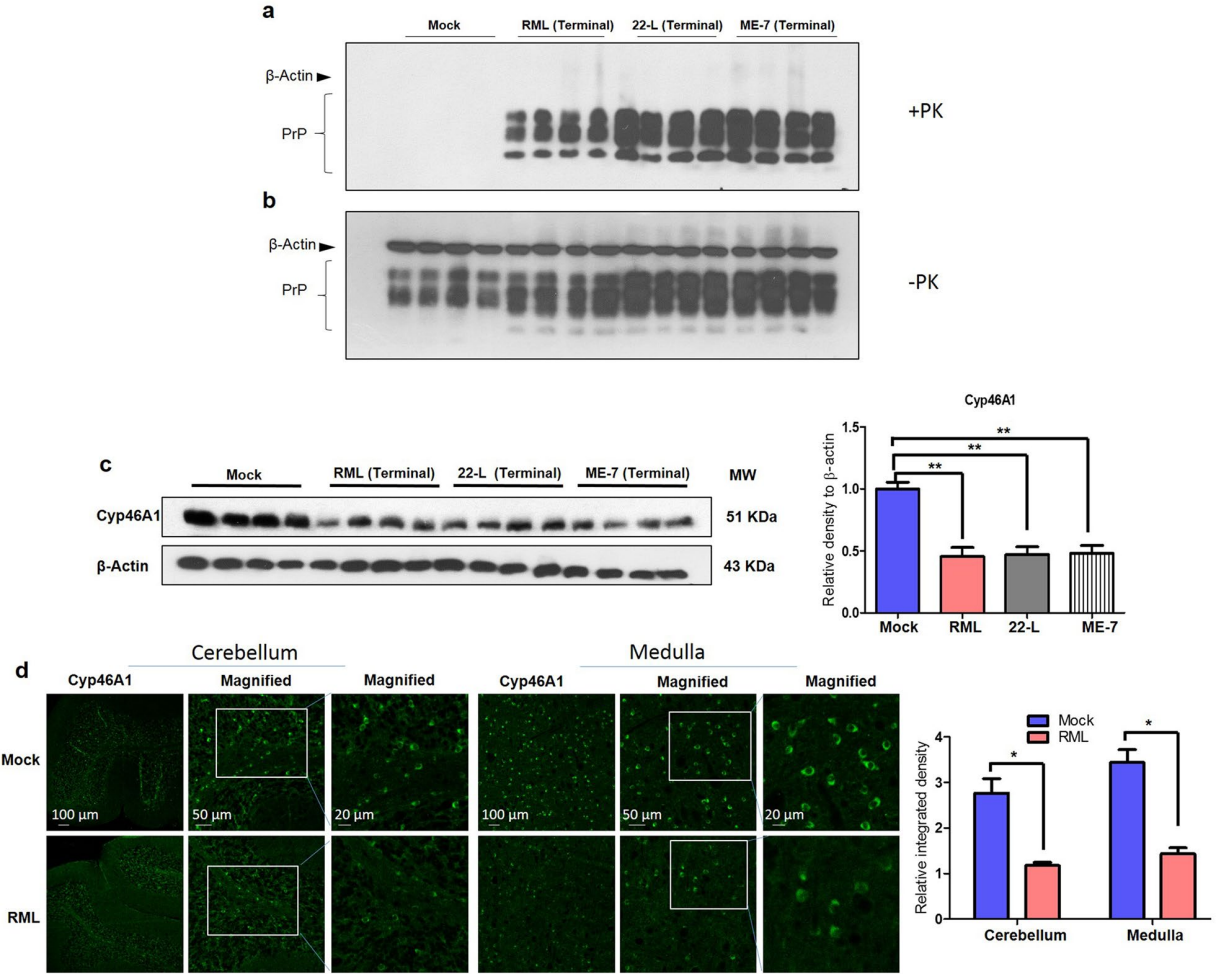
Reduced Cyp46A1 protein levels at terminal stages of prion disease in mice. **a**, **b** PK-digested (PK +) and undigested (PK-) brain homogenates of mock and prion-infected mice (RML, 22-L and ME-7) were used for immunoblotting and PrP detection using mAb 4H11. No PK-resistant PrP^Sc^ (PrP^res^) was detected in + PK samples in the mock group, while PrP^Sc^ was abundantly detected in all prion-infected brain homogenates. **c** The same brain homogenates of mock and prion-infected mice (RML, 22-L and ME-7) at terminal stage (> 150 days post infection (DPI) were analyzed by immunoblot for Cyp46A1 levels. β-Actin was used as a loading control and was obtained after stripping of the same membrane. The histograms are represented as the means ± SEM (*n* = 4 mice/group) of three independent experiments. Significance = **p < 0.01; ANOVA followed by Turkey’s post hoc test. **d** Immunofluorescence images of Cyp46A1 (green) in the cerebellum and medulla regions of mouse brains. Fluorescence intensity was quantified; the histograms represent the means ± SEM of *n* = 3 mice per group, obtained from 3 independent experiments. Magnification: 10X. Scale bar = 100 μm, 50 μm, 20 μm. Significance = *p < 0.05; Student’s unpaired t test

To assess a potential role of Cyp46A1 in prion disease, we analyzed its protein levels at the terminal stage of prion disease after intracerebral (i.c.) inoculation of three well established scrapie strains (RML, 22L and ME7) into FVB mice. The mice were closely monitored for progression of clinical signs of prion disease and animals were euthanized at terminal stage, along with non-infected mock groups of mice. We confirmed prion infection by immunoblot analysis of PrP^Sc^ levels upon proteinase K (PK) digestion of brain homogenates. Results indicated strong signals for PrP^Sc^ in all RML-, 22L- and ME7-infected FVB mice, while in non-infected (mock) animals we did not detect any PrP^Sc^ signal (Fig. 1a, b). Following this confirmation, we assessed the Cyp46A1 levels in the same brain homogenates of RML, 22L and ME7-infected and non-infected mice, respectively, by immunoblot. Cyp46A1 signals were quantified, and the results indicate a significant reduction of Cyp46A1 in brain homogenates at the terminal stage of prion disease (RML, 22L, and ME7; *p* < 0.01) compared to the age-matched non-infected mice (Fig. 1c; *p* < 0.01). We also found reduced Cyp46A1 immunofluorescence reactivity in the brains of infected mice as compared to the non-infected group (Fig. 1d; *p* < 0.05).

These results led us to investigate the levels of Cyp46A1 at pre-symptomatic and early clinical stages of prion disease. Therefore, separate cohorts of mice were euthanized at specified periods of time after i.c. infection with RML, 22L and ME7 prions, such as 125 days post infection (DPI), 100 DPI and 75 DPI, along with corresponding non-infected (mock) mice. While at 75 and 100 DPI the mice were at a pre-clinical stage of disease, at 125 DPI early prion signs manifested, such as a slight to moderate rigid tail, and a slightly hunched posture. At all-time points, PK-resistant PrP^Sc^ was detectable in brain homogenates of all infected mice (Additional file 1: Fig. S1ab, S2a, b & S3a, b). However, no significant differences in Cyp46A1 expression levels were measurable by immunoblot (Additional file 1: Fig. S1c, S2c & S3c).

**Fig. 2:**
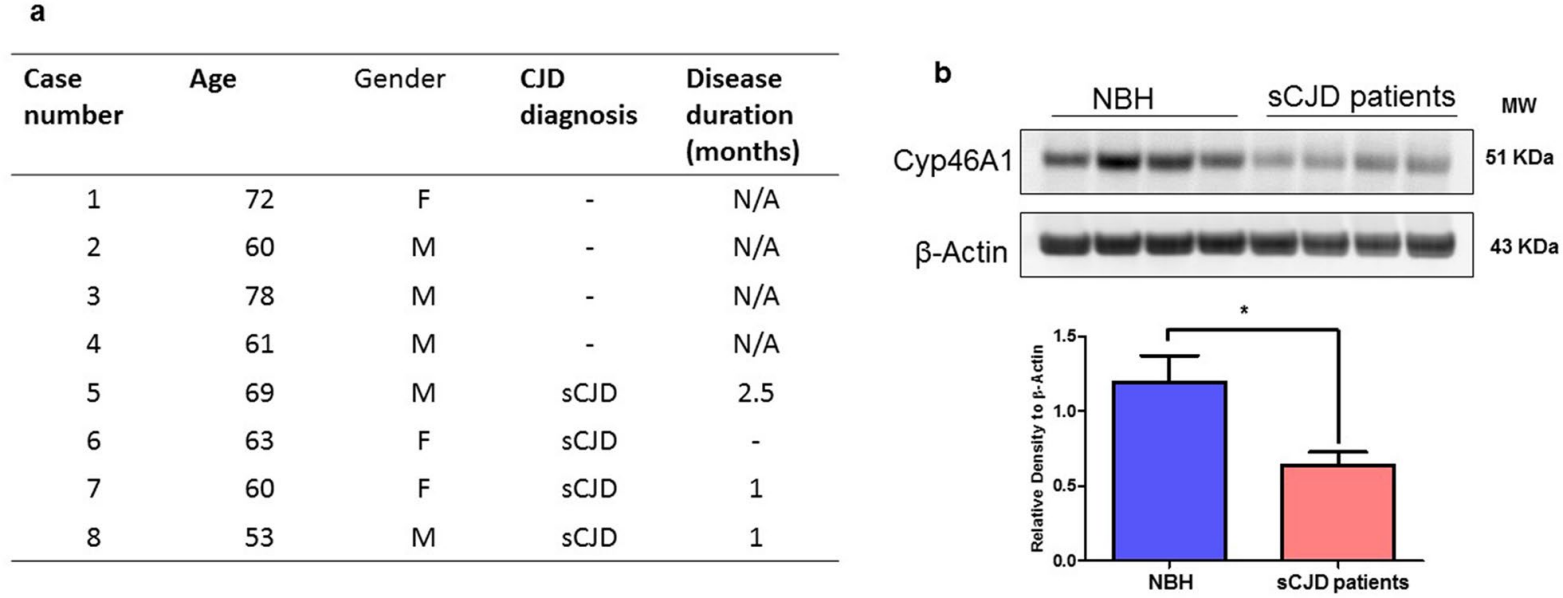
Reduced Cyp46A1 levels in human sCJD brains. **a** Demographic details of the post-mortem brain of four different healthy individuals (NBH: normal brain homogenates) and four different sporadic CJD patients (sCJD). **b** Cyp46A1 levels in the brain homogenates of healthy individuals (NBH) and sCJD patients. β-Actin was used as a loading control and was obtained after stripping of the same membrane. The histogram represents the means ± SEM (*n* = 4 humans/group) of three independent experiments. Significance = *p < 0.05; Student’s unpaired t test

**Fig. 3:**
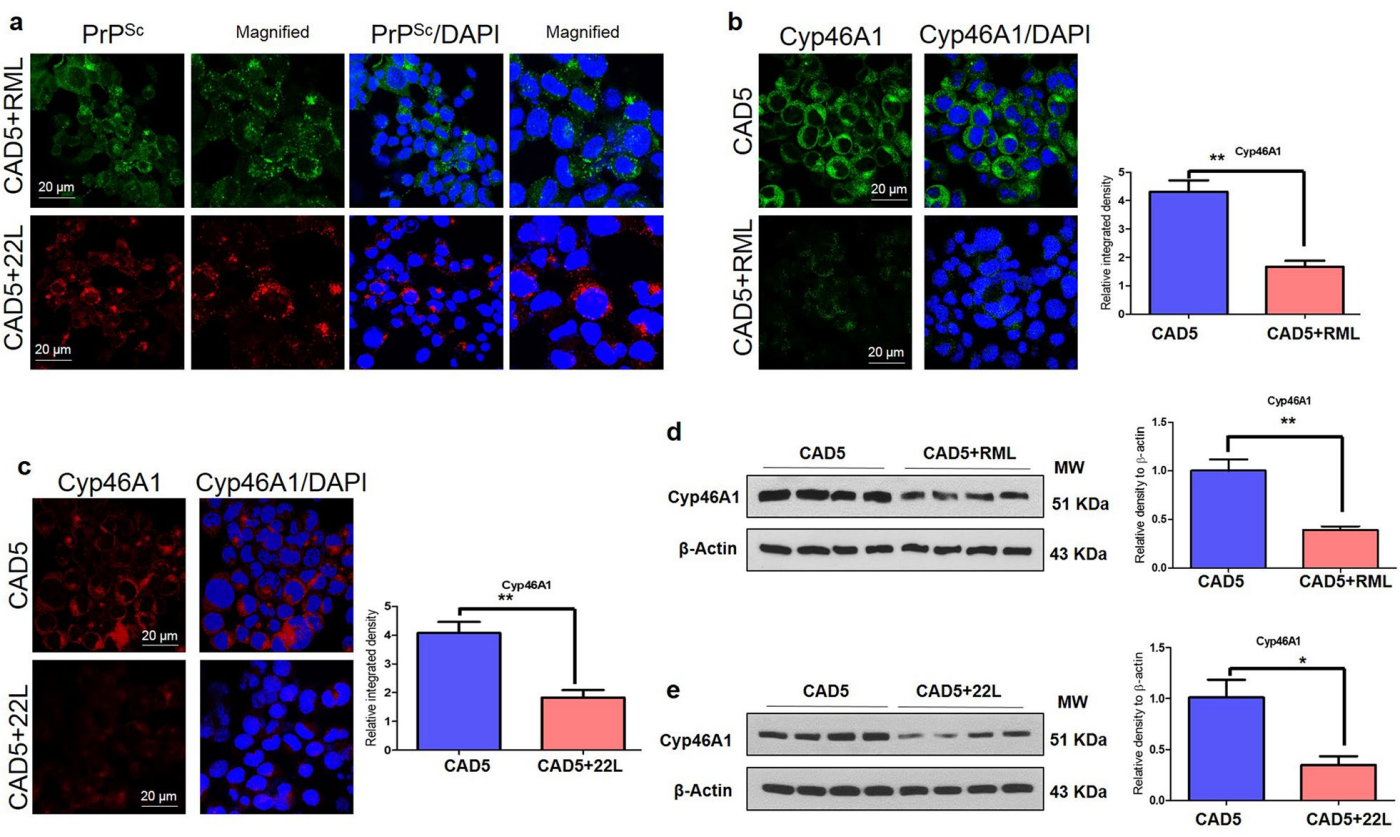
Cyp46A1 levels in prion-infected neuronal cells. **a** Immunofluorescence images of PrP^Sc^ (Green: Alexa Fluor 488; Red: Alexa Fluor™ 555; Blue: DAPI) for CAD5-RML and CAD5-22L. Magnification: 63X. Scale bar = 20 µm. **b**,** c** Immunofluorescence analysis of Cyp46A1 (Green: Alexa Fluor 488; Blue: DAPI) for CAD5 and CAD5-RML (Red: Alexa Fluor™ 555; Blue: DAPI) and for CAD5 and CAD5-22L cells. Fluorescence intensity was quantified; data are indicated as the mean ± SEM for *n* = 5 images per group from 3 independent experiments. Magnification: 63X. Scale bar = 20 µm. Significance = *p < 0.05; Student’s unpaired t test. **d**, **e** Immunoblot results of Cyp46A1 in CAD5, CAD5-RML and CAD5-22L cells. β-Actin was used as a loading control and was obtained after stripping the same membrane. The histograms depict the means ± SEM of three independent experiments. Significance = **p < 0.01; Student’s unpaired t test

To confirm results in the most common human prion disease and in order to determine the translational significance of Cyp46A1 reduction in prion-infected mice, post-mortem brain homogenates of four different healthy individuals (NBH: normal brain homogenates) and four different sporadic CJD patients (sCJD) (Fig. 2a) were analyzed by immunoblot for Cyp46A1 levels. Of note, as in the experimental models of prion disease, a significant reduction of Cyp46A1 levels in the brains of sCJD patients as compared to the healthy individuals was observed, confirming the relevance of our finding to human prion diseases (*p* < 0.05).

### Cyp46A1 is reduced in neuronal cells persistently infected with prions

Next, in vitro experiments were performed to confirm that the reduced level of Cyp46A1 in animal models and human sCJD patients was associated with prion infection. We used the mouse catecholaminergic neuronal cell line CAD5 persistently infected with RML and 22L prions (CAD5-RML; CAD5-22L). Prion infection was confirmed by immunofluorescence assay using conditions for the specific detection of PrP^Sc^ (Fig. 3a). Next, we performed confocal microscopy for assessing Cyp46A1 immunofluorescence reactivity. The confocal microscopy results indicated significantly reduced Cyp46A1 immunofluorescence reactivity in the RML- or 22L-infected CAD5 neuronal cells (CAD5-RML; CAD5-22L) as compared to the uninfected CAD5 neuronal cells (Fig. 3b, c; *p* < 0.01; *p* < 0.01). These results were verified by immunoblot. Of note, we found significantly reduced Cyp46A1 levels in CAD5-RML and CAD5-22L cells as compared to the uninfected CAD5 neuronal cells (Fig. 3d, e; *p* < 0.01; *p* < 0.05).

The reduction of Cyp46A1 we observed both in vivo and in vitro indicates that Cyp46A1 has a role in prion diseases. Overall, our results suggest that increasing Cyp46A1 activity in prion diseases can be a novel therapeutic approach in prion diseases.

### EFV treatment reduced PrP^Sc^ in prion infected neuronal cells

**Fig. 4:**
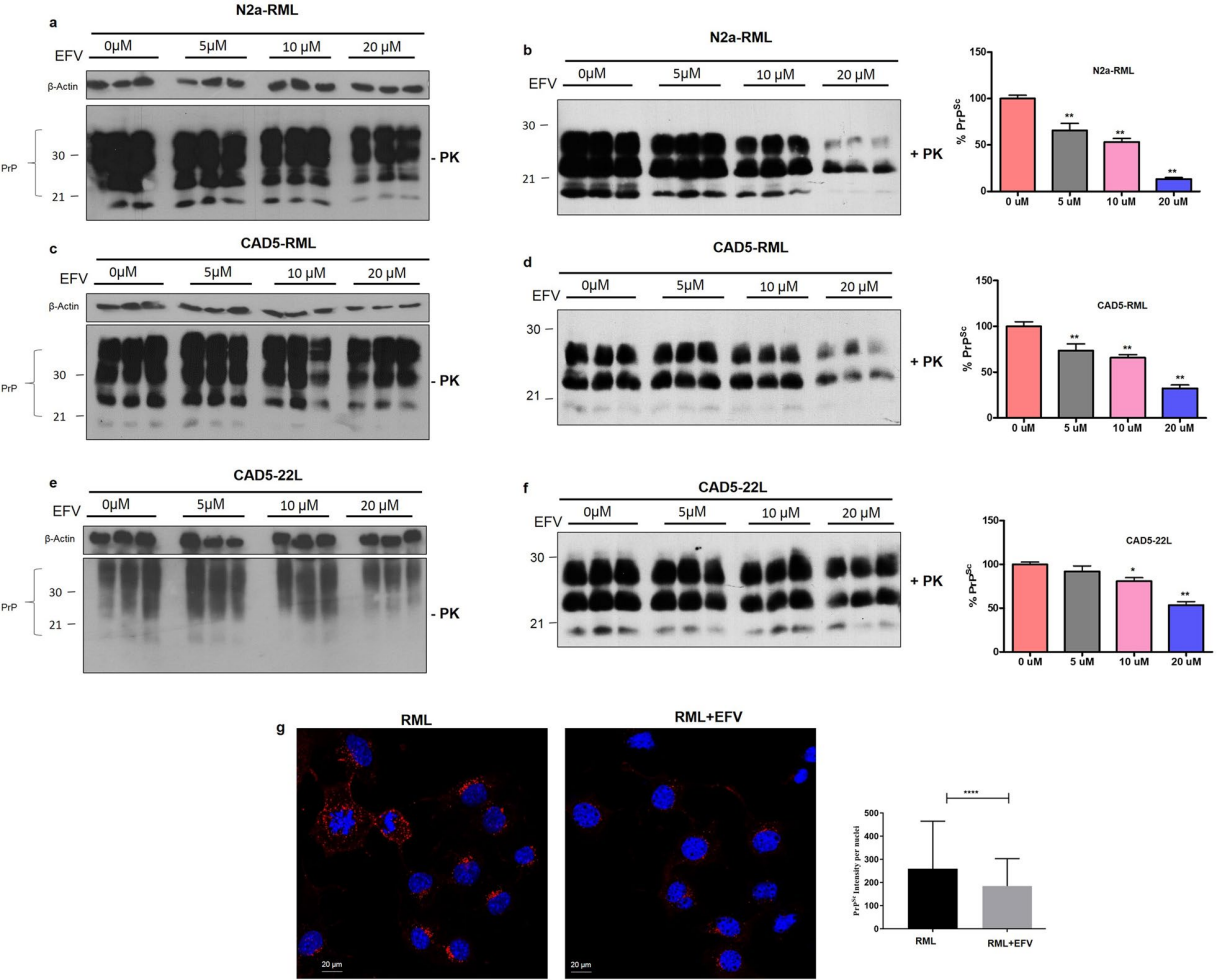
EFV treatment reduced PrP^Sc^ in prion infected neuronal cells. Immunoblot images of PrP signals in cell lysates of RML-infected N2a cells (**a**,** b**), RML-infected CAD5 cells (**c**, **d**) and 22L-infected CAD5 cells (**e**, **f**) treated with EFV (5 µM, 10 µM, 20 µM) or vehicle (0 µM) for three days. For PrP^Sc^ detection, samples were digested with PK (+ PK). PrP was detected using mAb 4H11. β-actin served as a loading control. The densitometric analysis of PrP^Sc^ signals (+ PK) is shown as a percentage of the signals in the untreated control cells. The data are indicated as the mean ± SEM for *n* = 3 per group, and the number of independent experiments = 3, each performed in triplicate. Significanc e = **p < 0.001; One-way ANOVA with Dunnett’s multiple comparison test. **g** Immunofluorescence images of PrP^Sc^ (Red: TRITC; Blue: DAPI) in RML-N2a cells treated with or without EFV (20 µM). The histogram represents the means ± SEM of the intensity of PrP^Sc^ staining/nuclei obtained from 3 independent experiments. Magnification: 63X. Scale bar = 20 µm. Significance = ****p < 0.0001; Student’s unpaired t test

Next, we aimed to use the antiretroviral drug and allosteric Cyp46A1 activator EFV as a potential therapeutic. EFV via activation of Cyp46A1 has a significant beneficial effect on the regulation of impaired brain cholesterol metabolism [36–41]. We used different neuronal cell lines (N2a and CAD5) infected with RML or 22L prions to assess a potential inhibitory effect of EFV treatment on prion propagation. To this end, N2a-RML, CAD5-RML and CAD5-22L cells were treated with EFV at different nontoxic concentrations (5 µM, 10 µM, 20 µM) for 3 days. Cells treated with EFV solvent served as a control. The cell lysates were subjected to proteinase K (+ PK) digestion or not and used for the analysis of PrP signals by immunoblot. The immunoblot results and quantification of the PK-digested samples indicated that EFV treatment significantly reduced the PrP^Sc^ level in N2a-RML, CAD5-RML and CAD5-22L cells in a concentration dependent manner as compared to the non-treated control cells. Analysis of cell lysates without PK digestion using anti-PrP antibody 4H11 reflected the decrease of PrP^Sc^ levels by a reduction of total PrP (Fig. 4a–f; *p* < 0.01; < 0.05). Further, we confirmed these findings by confocal microscopy upon PrP^Sc^-specific staining of prion-infected cells. The results revealed that EFV treatment significantly reduced the immunofluorescence reactivity of PrP^Sc^ in RML-N2a cells compared to non-treated control cells (Fig. 4g; *p* < 0.0001).

Next, we tested the efficacy of EFV treatment to reduce PrP^Sc^ propagation in a more physiological model, namely prion-infected primary cerebellar granular neuronal (CGN) cultures. CGNs prepared from newborn C57Bl/6 mice were infected with 22L prions. EFV (5 µM, 10 µM) was added twice a week, starting at day 1 (D1) and day 7 (D7), respectively, after infection. Cells were lysed at different time points after infection. Of note, in the prion-infected primary CGN culture, EFV treatment also reduced the PrP^Sc^ levels as observed by immunoblot analysis of PK digested cell lysates. This effect was particularly evident on day 21 post infection when EFV treatment was started at 1 day post infection at a concentration of 10 µM (Additional file 1: Fig. S4). Overall, these results show that EFV treatment has a strong anti-prion effect in neuronal cell lines and primary neuronal cells which is neither dependent on prion strain nor cell line specific, proposing EFV as a potent therapeutic in prion diseases.

### EFV treatment does not affect PrP^C^ level and lipid raft integrity in neuronal cells

**Fig. 5:**
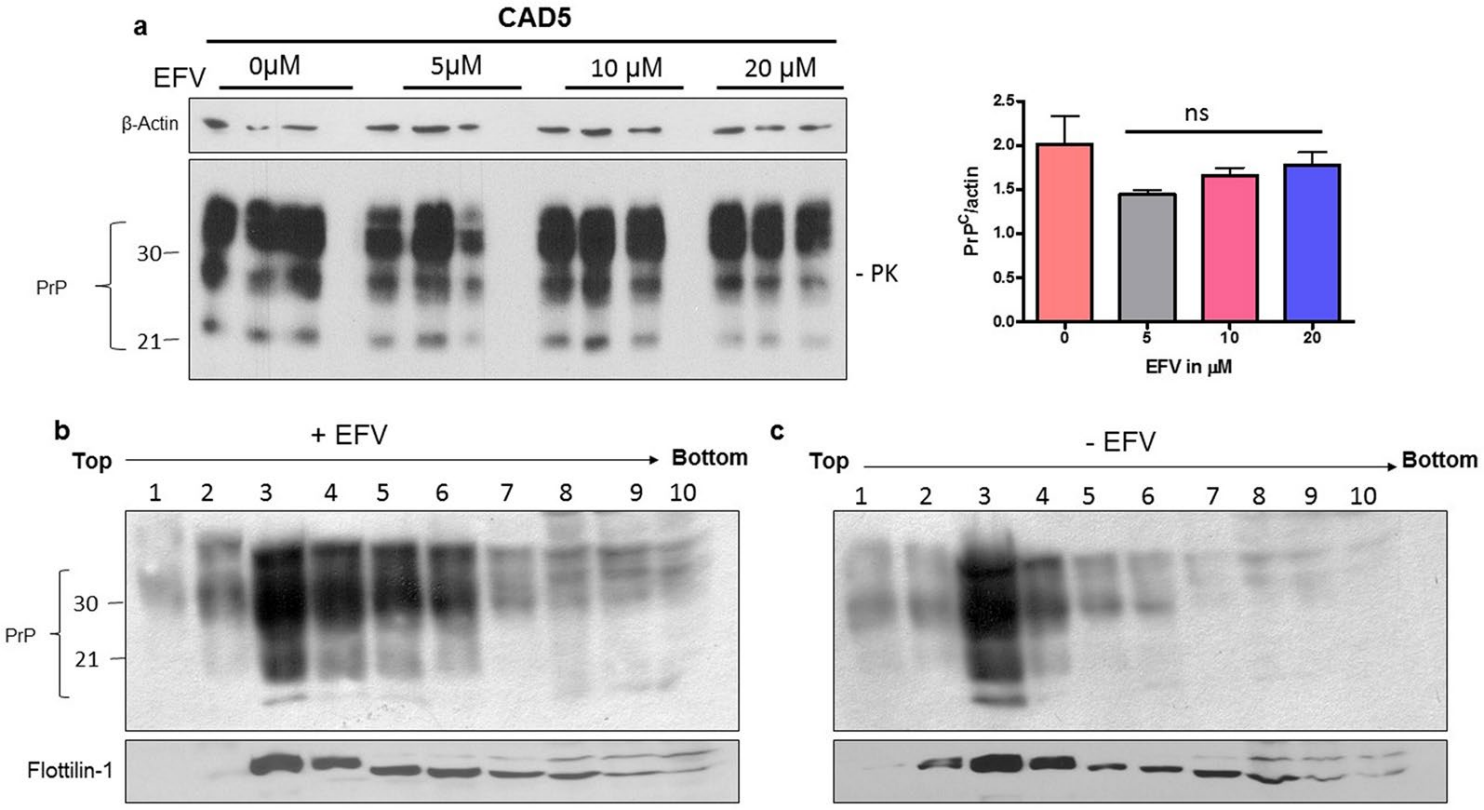
The effect of EFV treatment on PrP^C^ level and lipid raft association in neuronal cells. **a** Non-infected CAD5 cell were treated with EFV (5 µM, 10 µM, 20 µM) or vehicle and PrP^C^ in cell lysates was detected by immunoblot using mAb 4H11. β-Actin was used as a loading control. The data are indicated as the mean ± SEM for *n* = 3 per group, and the number of independent experiments = 3, each performed in triplicate. Significance = no significant (ns) differences **b**,** c** N2a-WT cells treated with EFV (+ EFV) or not (-EFV) were lysed in cold Triton-X 100 and subjected to flotation density gradients. Fractions 1–10 were collected from the top to the bottom of the gradient and analyzed by immunoblot using anti-PrP antibody 4H11 and anti-flotillin-1 as a lipid raft marker. **b** Representative immunoblot result of PrP in the fractions of cells treated with EFV. **c** Representative immunoblot result of PrP in the fractions of cells without EFV treatment

Manipulation of cellular cholesterol turnover can affect lipid raft integrity and PrP^C^ trafficking or amount. Therefore, we used non-infected CAD5 neuronal cells to determine effects of EFV treatment on PrP^C^. The CAD5 neuronal cells were treated with the same concentrations (5 µM, 10 µM, 20 µM) of EFV or with vehicle for 3 days as used in the prion-infected cells. The immunoblot results of cell lysates showed that there was no significant difference in total PrP^C^ levels in the EFV-treated groups at all concentrations as compared to the control group (Fig. 5a).

Primarily, PrP^C^ is localized at the plasma membrane and particularly in lipid rafts, which is important for the physiological functions of PrP^C^ [9–14]. Therefore, we used CAD5 cells treated with or without EFV to analyze the association of PrP^C^ with lipid rafts. A density gradient procedure was applied according to previous protocol [11, 12] for separation of lipid raft domains. Fractions of the gradient were analyzed by immunoblot for PrP^C^ and flotillin, a marker for lipid rafts. This revealed that highest levels of PrP^C^ existed in fractions 3–6, coherent with the signals of flotillin in fractions 3–6. In EFV treated cells, PrP^C^ and flotillin co-fractionated and were mainly found in the same fractions as in non-treated cells (Fig. 5b, c).

These results indicated that EFV treatment has no adverse effect on the total PrP^C^ level. Lipid raft integrity was maintained upon EFV treatment and lipid raft association of PrP^C^ was not affected.

### Oral EFV treatment delays prion disease and extends survival times of prion-infected animals

**Fig. 6:**
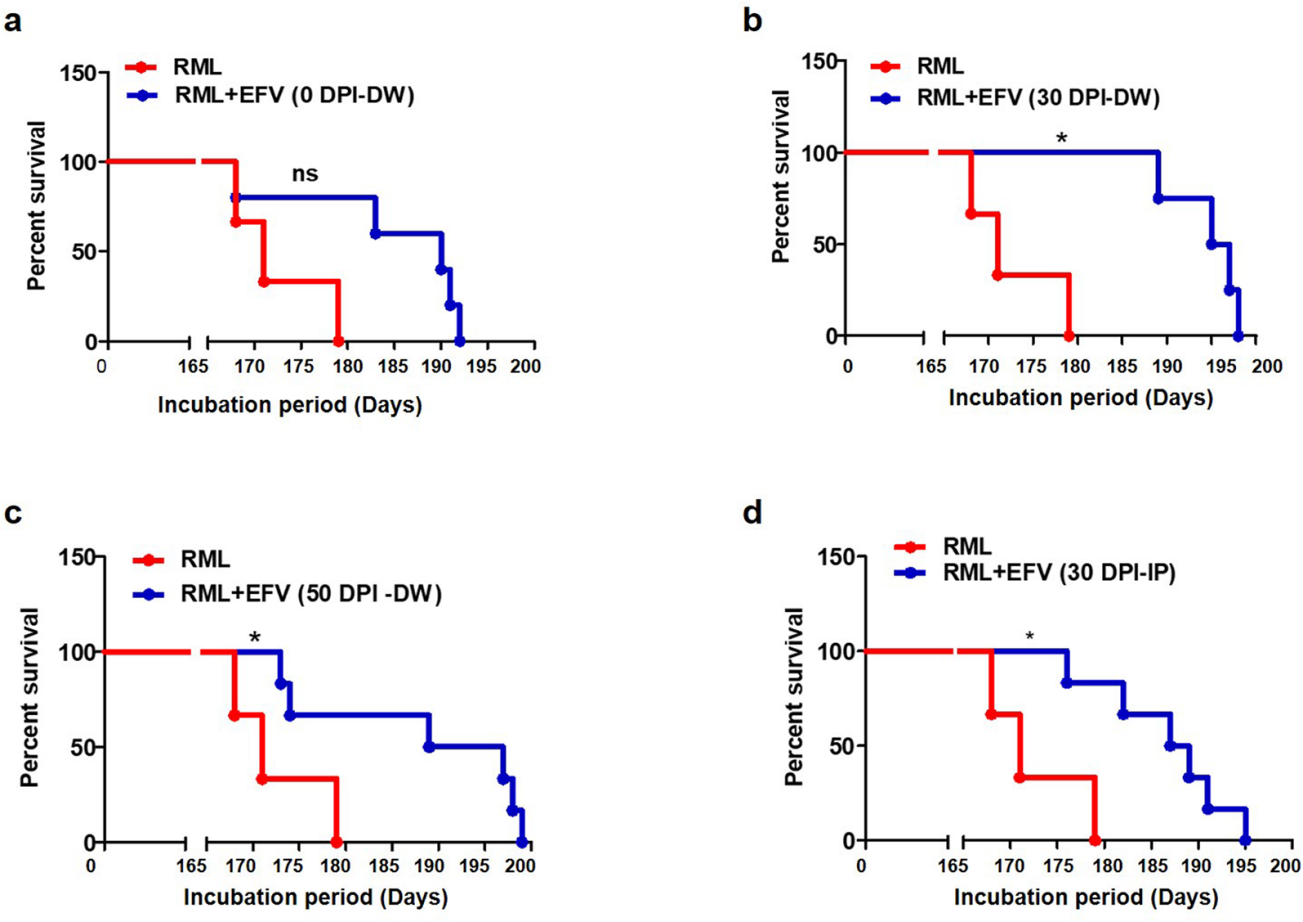
Oral EFV treatment delays prion disease and extends survival of prion-infected animals. **a** The representative Kaplan–Meier plot indicates the percent survival of RML-infected mice in the untreated group (*n* = 9 mice) and EFV-treated group RML + EFV (0 DPI-DW) (*n* = 7 mice), using log rank test for statistical analysis. **b** The representative Kaplan–Meier plot indicates the percent survival of RML-infected mice in the untreated group (*n* = 9 mice) and EFV-treated group RML + EFV (30 DPI-DW) (*n* = 9 mice), using log rank test for statistical analysis. **c** The representative Kaplan–Meier plot indicates the percent survival of RML-infected mice in the untreated group (*n* = 9 mice) and EFV-treated group RML + EFV (50 DPI-DW) (*n* = 8 mice), using log rank test for statistical analysis. **d** The representative Kaplan–Meier plot indicates the percent survival of RML-infected mice in the untreated group (*n* = 9 mice) and EFV-treated group RML + EFV (30 DPI-IP) (*n* = 7 mice), using log rank test for statistical analysis. NS *p* > 0.05, *p < 0.05. The x-axes depict the days post i.c. infection with RML prions

The significant inhibitory effects on PrP^Sc^ propagation of the Cyp46A1 activator EFV in in vitro models of prion infection, and the reduced expression of Cyp46A1 in prion-infected mice, sCJD patients and prion-infected neuronal cells, inspired us to evaluate EFV effects in an animal model of prion disease. The EFV treatment was selected at the lowest dose (0.09 mg/kg/day) previously published to activate Cyp46A1 in mouse brains, and is 300–400-fold lower than that used for chronic treatment of HIV patients [19, 41]. In one treatment paradigm, we started the low dose (0.09 mg/kg/day) EFV treatment applied orally in drinking water on the same day (0 DPI), at 30 DPI or at 50 DPI of intracerebral infection of C57Bl/6 mice with RML prions. Treatment was continued until the experimental endpoint at which mice reached the terminal stage of prion disease [19, 41]. The survival times of the EFV-treated mice (RML + EFV (0 DPI-DW) were increased when compared with untreated, RML-infected control mice [RML, 170.9 ± 3.430; RML + EFV (0 DPI-DW), 182.3 ± 3.496] and the mean of the extended survival time of treated mice was 11.40 ± 4.968 days compared with RML-infected mice (Fig. 6a).

Interestingly, when we started EFV treatment at 30 or 50 DPI, the survival time of the mice was even more extended. The survival times in both treatment groups were significantly increased compared with untreated, RML-infected control mice [Fig. 6b; RML 170.9 ± 3.430; RML + EFV (30 DPI-DW), 189.6 ± 2.489)]; [Fig. 6c; RML 170.9 ± 3.430; RML + EFV (50 DPI-DW), 187.0 ± 3.698]. The mean extended survival time of mice revealed a significant prolongation of 18.67 ± 4.238 [Fig. 6b; RML + EFV (30 DPI-DW)] and 16.11 ± 5.039 [Fig. 6c; RML + EFV (50 DPI-DW)] days, respectively, compared to the non-treated and infected control group.

In order to achieve more control on the drug dosage received by each mouse, we included another treatment paradigm for one group of mice. We administered EFV intraperitoneally (I.P) starting on day 30 post infection at a concentration of 0.05 mg/kg three times a week until 130 DPI. Due concerns of stress induced by continuous I.P injections, after 130 DPI we continued with EFV treatment (0.09 mg/kg/day) supplied in drinking water until the end of the experiment. Notably, in this treatment group the survival times were also significantly increased compared with untreated, RML-infected control mice [Fig. 6d; RML, 170.9 ± 3.430; RML + EFV (30 DPI-IP, 185.8 ± 2.111)]. The mean extended survival times of mice were significantly prolonged by 14.86 ± 4.154 days as compared to RML-infected and untreated mice (Fig. 6d). These results unveiled that as the prion disease progressed towards the terminal stage, EFV treatment induced a significant prolongation of survival of prion-infected animals.

## Discussion

The failure and disappointing outcomes of clinical trial to treat human prion diseases and the potential risk of zoonosis urges research to identify novel therapeutic targets to treat these deadly diseases. To the best of our knowledge, our study provides the first evidence of significantly decreased levels of Cyp46A1 in prion-infected neuronal cells, in brains of mice infected with different prion strains at the terminal disease stage and most importantly, in post-mortem brains of sCJD patients. In brains of mice or humans with terminal prion disease Cyp46A1 might be reduced because of the loss of neurons; however, our findings of lower Cyp46A1 levels in prion-infected neuronal cells that do not exhibit signs of cell death as compared to non-infected cells indicate an association with prion infection. Our finding of Cyp46A1 downregulation is one among other examples showing a specific protein that is affected in prion disease [48, 49].

Cholesterol is synthesized locally in the brain, thereby cholesterol homeostasis and metabolism depend on local cholesterol elimination. Notably, the BBB does not allow cholesterol to cross from the brain to the peripheral circulation. The cholesterol elimination rate depends on Cyp46A1, a brain specific enzyme which converts cholesterol into 24-OHC, a molecule which can easily permeate the BBB, and reach the peripheral circulation system for further metabolism [21, 50]. Cyp46A1 is a member of the cytochrome 450 enzymes family and distributed through different regions of the brain such as hippocampus, cortex, striatum, thalamus and cerebellum [51]. It is primarily expressed in neuronal cells and is considered the main enzyme with a primary role in neuronal cholesterol metabolism [52, 53]. Reduced levels of CYP46A1 have been associated with other protein misfolding diseases such as Alzheimer’s disease (AD), Parkinson’s disease (PD) and Huntington’s disease (HD) as well as other neurodegenerative diseases e.g. Niemann-Pick disease type C (NPC) [17–21]. On the other hand, the overexpression and pharmacological activation of Cyp46A1 induce beneficial effects and rescue brain disorders [36–41]. The Cyp46A1 metabolite 24-OHC is a local, non-toxic and neuroprotective metabolite acting as an activator of liver X receptors, which have a key role in the functionality of ABCA1 and other cholesterol efflux genes (apolipoproteins) in both neuronal and glial cells which subsequently regulate brain cholesterol homeostasis and turnover. The 24-OHC is responsible for regulating the activity of various transcription factors and mediators e.g. sterol regulatory element-binding protein 2, involved in cholesterol homeostasis in the brain [31–34]. Further, Cyp46A1 is contributing to protein phosphorylation and ubiquitination as well as to many cellular signaling pathways and events such as endo-lysosomal trafficking, autophagy, synaptic trafficking and neuronal survival signaling such as brain derived neurotropic factor (BDNF) and TrkB receptor signaling [17–21, 53, 54]. Our results, together with evidence from other studies indicate a role of Cyp46A1 in aberrant brain cholesterol metabolism in neurodegenerative diseases and that it would be a valuable and novel drug target in prion diseases.

Interestingly, in vitro and in vivo findings demonstrated that neuronal cholesterol synthesis and amount are increased in prion infection [9–12, 26], suggesting that elevated cholesterol might be a contributing factor in the pathogenesis of prion disease. Our findings of reduced Cyp46A1 levels in prion-infected neuronal cells and in vivo in mouse brains and post-mortem brain homogenates of sCJD patients provide an explanation since the rate of cholesterol export from the brain will be affected by a reduction of Cyp46A1. Upon prion infection, ABCA1 is displaced from lipid rafts and the cell surface and internalized to intracellular compartments leading to a loss of function. ABCA1 is one of the complementary pathways responsible for neuronal cholesterol efflux. Thus, prion infection interferes with neuronal cholesterol efflux through reduction of Cyp46A1 and deregulation of ABCA1 function. Since ABCA1 is regulated by Cyp46A1 and 24-OHC, its deregulation might be a consequence of Cyp46A1 reduction. Eventually, the impairments of cholesterol efflux pathways upon prion infection lead to an increase and accumulation of cholesterol [24–26]. Such high cholesterol in membranes might favor PrP^Sc^ replication. Thereby, the process of PrP^C^ conversion into PrP^Sc^ resulting in alleviation of cholesterol elimination might continue as a vicious cycle implicated in the progression of prion disease. Therefore, it is required to explore mechanisms through which aberrant neuronal cholesterol metabolism can be regulated to halt the propagation of PrP^C^ to PrP^Sc^ and subsequently the severity of prion disease.

In line with this, we demonstrate here that Cyp46A1 activation represents a novel and efficient therapeutic strategy against prion diseases. We used treatment with EFV, an antiretroviral drug known to activate Cyp46A1 and achieved a significant reduction of PrP^Sc^ propagation in prion-infected neuronal cells and primary CGN cultures (Fig. 4a–g; Additional file 1: Fig. S4). The exact mechanism by which PrP^C^ converts into PrP^Sc^ is not comprehensively known. On a subcellular level, a large body of evidence indicates that this conversion occurs in lipid rafts of the plasma membrane. Lipid rafts are membrane microdomains enriched in cholesterol and sphingolipids and harbor many glycosyl phosphatidyl inositol (GPI)-anchored proteins. Both PrP^C^ and PrP^Sc^ are GPI-anchored proteins and found in lipid rafts. It has been shown that PrP^Sc^ propagation can be prevented by reducing cellular cholesterol, e.g. by treatment with statins, cyclodextrin or amphotericin B, thereby destroying the integrity of lipid raft microdomains [55, 56]. We reported that cholesterol is required for the distribution and localization of PrP^C^ at the cell surface, a pre-requisite for propagation of PrP^Sc^ [23, 24]. Treatment with many of these cholesterol-lowering drugs interferes with cellular PrP^C^ content and its lipid raft association, raising concerns about compromised function of PrP^C^ in signaling pathways. Notably, EFV treatment neither affects PrP^C^ levels nor its lipid raft association. Rather than resulting in cholesterol depletion, EFV treatment enhances cholesterol turnover at the plasma membrane [21, 38, 57], suggesting an alternative, novel anti-prion mechanism that does not primarily affect PrP^C^.

Oral treatment of prion-infected mice significantly prolonged the survival of animals and delayed the progression of prion disease (Fig. 6a–d). EFV is an allosteric activator of Cyp46A1. It crosses the BBB and has been used for the chronic treatment of HIV patients for decades, at much higher doses as used here. Several lines of evidence showed that oral EFV treatment rescues memory deficits, reduces amyloid beta and extends the survival of animals [19, 37–41]. In addition, EFV treatment, through regulation of impaired cholesterol metabolism, reduced misfolded proteins e.g. p-tau and amyloid beta, two main hallmarks in AD [58]. Very recently, two studies reported that EFV enhanced the lifespan of mice with brain tumor and in NPC disease [19, 41]. Mitroi et al. further confirmed that EFV activates Cyp46A1 and mobilizes it to the plasma membrane which is responsible for restoring cholesterol efflux and improving learning and memory functions in NPC disease animal models [19]. Recently, it was reported that Cyp46A1 overexpression and its activation by EFV, respectively, mediate sterol flux, significantly contribute to the plasma membrane properties and regulate various kinases such as cyclin-dependent kinase-5 and glycogen synthase kinase-3 which have a role in the formation of misfolding proteins e.g. p-tau and amyloid beta [57]. Our results demonstrate that EFV has significant beneficial effects in prion disease, likely associated with Cyp46A1 activation, eventually regulating impaired brain cholesterol metabolism. Our intriguing findings in mouse models of prion disease indicate that EFV treatment in all paradigms of oral application at a very low dosage significantly extended the survival times of prion-infected mice and delayed the progression of prion disease. Notably, this was achieved upon i.c. inoculation of mice, demonstrating that EFV exerted its effects in the brain. As a caveat, our studies were done using mouse-adapted scrapie strains and to corroborate the relevance of our findings we suggest further studies using infection models for human prions. Furthermore, the drug was delivered orally in drinking water, which introduces some individual variability with regards to the drug dose received by the mice, depending on their fluid intake. In HIV patients, EFV medication induced some undesirable side effects such as neuropsychiatric adverse effects [59]; however, in all of the previous pre-clinical animal studies and in our bioassay, EFV was applied at lowest doses (0.09 mg/kg/day, orally and 0.05 mg/kg, i.p., 3 times/week) and did not induce any type of side effects. The EFV dosage used here (0.09 mg/kg/day) is 300–400-fold lower than that used for HIV patients, activates Cyp46A1 in mice and has been very effective in all animal models of neurodegenerative diseases [19, 41]. Other experimental anti-prion drugs have been applied especially the statin drugs which inhibit the cholesterol biosynthesis and they were not effective to prevent the PrP^Sc^ accumulation and its associated pathologies [60, 61]. Hence, the ability of EFV to cross the BBB, the safety profile at low dose, and the efficacy and tolerability of chronic administration through the oral route indicate a strong translational potential.

In summary, our results demonstrate that regulation of brain cholesterol metabolism through pharmacological activation of Cyp46A1 using EFV is a promising approach for intervention in prion diseases.

## Supplementary Information

**Additional file 1**. Supplementary Information.

## Data Availability

All data required to validate our hypotheses in the paper are provided in the paper and/or the Supplementary Materials. Further any additional data related to this manuscript will be available upon request from the corresponding author.
