# Supplementary material for: Oral administration of repurposed drug targeting Cyp46A1 increases survival times of prion infected mice
Source: Acta Neuropathol Commun. 2021 Apr 1;9:58. doi: 10.1186/s40478-021-01162-1 (PMC8017635; doi:10.1186/s40478-021-01162-1)
Supplement: Supplementary file 1 — Additional file 1. Supplementary Information. [file 40478_2021_1162_MOESM1_ESM.docx]

**Supplementary Information**

**Oral administration of repurposed drug targeting Cyp46A1 increases survival times in preclinical trials towards treatment of prion disease**

Tahir Ali^1,2^, Samia Hannaoui^1,2^, Satish Nemani^3^, Waqas Tahir^1,2^, Irina Zemlyankina^1,2^, Pearl Cherry^1,2^, Su Yeon Shim^1,2^, Valerie Sim^3^, Hermann M. Schaetzl^1,2^, Sabine Gilch^1,2*^

^1^ Calgary Prion Research Unit, Department of Comparative Biology & Experimental Medicine, Faculty of Veterinary Medicine, University of Calgary, Calgary, Alberta, Canada.

^2^ Hotchkiss Brain Institute, Cumming School of Medicine, University of Calgary, Calgary, Alberta, Canada.

^3^ Department of Medicine - Division of Neurology, Centre for Prions and Protein Folding Diseases, University of Alberta, Edmonton, Alberta, Canada.

**Running title: Efavirenz prolongs survival of prion-infected mouse models**

^*^Corresponding author

Sabine Gilch, PhD

Associate Professor

Canada Research Chair in Prion Disease Research

Dept. of Comparative Biology & Experimental Medicine, Faculty of Veterinary Medicine

University of Calgary, 3330 Hospital Drive NW Calgary, AB T2N 4Z6, Canada

Office: HRIC1AC66; Phone: (403) 210-7578

E-mail: [sgilch@ucalgary.ca](mailto:sgilch@ucalgary.ca)

**Table S1. Animal bioassay II**

| **Mouse ID** | **RML Inoculation** | **Groups** | **Drug Treatment** | **DPI** | **Mouse Euthanization, Terminal**  **Deceased/Humane Endpoints** |
| --- | --- | --- | --- | --- | --- |
| 2CsR-02925 | - | **RML** | **No treatment** | 186 | Terminal |
| 2CsR-02926 | - | - | - | 162 | Terminal |
| 2CsR-02927 | - | - | - | 162 | Terminal |
| 2CsR-02928 | - | - | - | 171 | Terminal |
| 2CsR-02929 | - | - | - | 186 | Terminal |
| 2CsR-02930 | - | - | - | 162 | Terminal |
| 2CsR-02931 | - | - | - | 168 | Terminal |
| 2CsR-02932 | - | - | - | 179 | Terminal |
| 2CsR-02933 | - | - | - | 164 | Deceased |
| 2CsR-02934 | - | - | - | 162 | Terminal |
| 2CsR-02915 | - | **RML+ 0 DPI (DW)** | **EFV** | 176 | Terminal |
| 2CsR-02916 | - | - | - | 183 | Terminal |
| 2CsR-02917 | - | - | - | 176 | Terminal |
| 2CsR-02918 | - | - | - | 190 | Terminal |
| 2CsR-02919 | - | - | - | 156 | Humane endpoint |
| 2CsR-02920 | - | - | - | 192 | Terminal |
| 2CsR-02921 | - | - | - | 168 | Terminal |
| 2CsR-02922 | - | - | - | 169 | Humane endpoint |
| 2CsR-02923 | - | - | - | 165 | Humane endpoint |
| 2CsR-02924 | - | - | - | 191 | Terminal |
| 2CsR-02905 | - | **RML+30 DPI (DW)** | - | 195 | Terminal |
| 2CsR-02906 | - | - | - | 193 | Terminal |
| 2CsR-02907 | - | - | - | 189 | Terminal |
| 2CsR-02908 | - | - | - | 179 | Terminal |
| 2CsR-02909 | - | - | - | 198 | Terminal |
| 2CsR-02910 | - | - | - | 165 | Humane endpoint |
| 2CsR-02911 | - | - | - | 183 | Terminal |
| 2CsR-02912 | - | - | - | 179 | Terminal |
| 2CsR-02913 | - | - | - | 193 | Terminal |
| 2CsR-02914 | - | - | - | 197 | Terminal |
| 2CsR-02885 | - | **RML+50 DPI (DW)** | - | 199 | Terminal |
| 2CsR-02886 | - | - | - | 183 | Humane endpoint |
| 2CsR-02887 | - | - | - | 197 | Terminal |
| 2CsR-02888 | - | - | - | 183 | Terminal |
| 2CsR-02889 | - | - | - | 183 | Terminal |
| 2CsR-02890 | - | - | - | 189 | Terminal |
| 2CsR-02891 | - | - | - | 174 | Terminal |
| 2CsR-02892 | - | - | - | 198 | Terminal |
| 2CsR-02893 | - | - | - | 183 | Humane endpoint |
| 2CsR-02894 | - | - | - | 173 | Terminal |
| 2CsR-02895 | - | **RML+30 DPI (PI)** | - | 189 | Terminal |
| 2CsR-02896 | - | - | - | 176 | Terminal |
| 2CsR-02897 | - | - | - | 183 | Terminal |
| 2CsR-02898 | - | - | - | 191 | Terminal |
| 2CsR-02899 | - | - | - | 195 | Terminal |
| 2CsR-02900 | - | - | - | 125 | Humane endpoint |
| 2CsR-02901 | - | - | - | 183 | Humane endpoint |
| 2CsR-02902 | - | - | - | 182 | Terminal |
| 2CsR-02903 | - | - | - | 133 | Deceased |
| 2CsR-02904 | - | - | - | 187 | Terminal |

**Supplementary figures and figure legends**

**
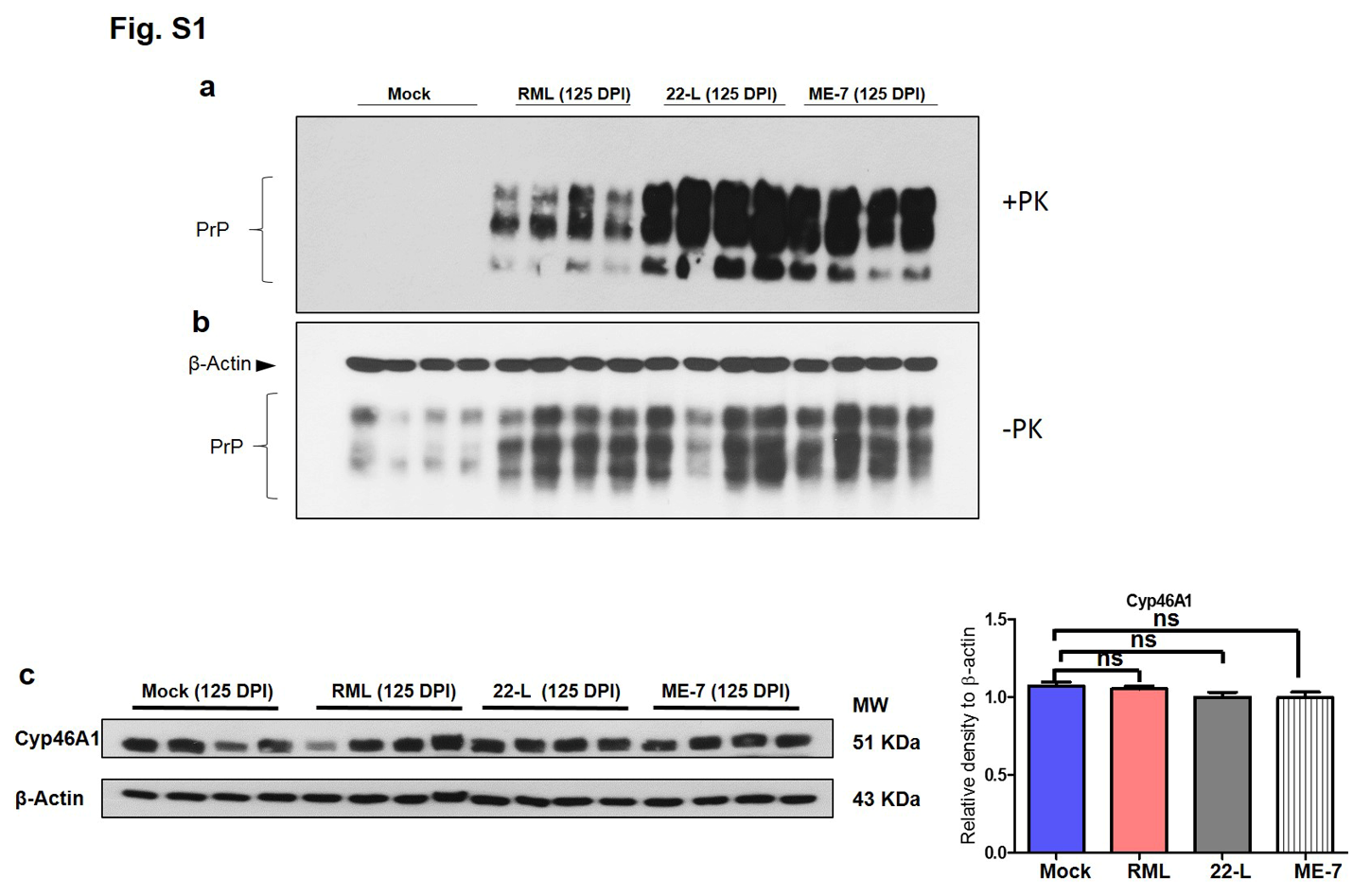
**

**Fig. S1.** **PrP and Cyp46A1 levels in mouse brain homogenates at 125 DPI.** **a, b** PrP levels at 125 DPI in brain homogenates of mice infected with different prion strains. PK-digested (PK+) and undigested (PK-) brain homogenates of mock and prion-infected (RML, 22-L and ME-7) mice were used for immunoblotting and detection of PrP using mAb 4H11. No PK-resistant PrP^Sc^ (PrP^res^) was detected in mock samples (+PK), while PrP^Sc^ was detected in all brain homogenates of prion infected mice at 125 DPI. β-actin was used as loading control. The PrP signal is high in all -PK samples at the expected ≈25–35 kDa range. In each case brain homogenates of 4 different mice for mock and RML, 22-L and ME-7 infection were used. **c** The same brain homogenates of mock and prion-infected mice (RML, 22-L and ME-7) at 125 DPI were analyzed by immunoblot for Cyp46A1 level. β-Actin was used as a loading control and was obtained after stripping the same membrane. Signals were quantified and the histograms represent the means ± SEM (n = 4 mice/group) of three independent experiments. Significance = no significance (ns); ANOVA followed by Turkey’s post hoc test.

**
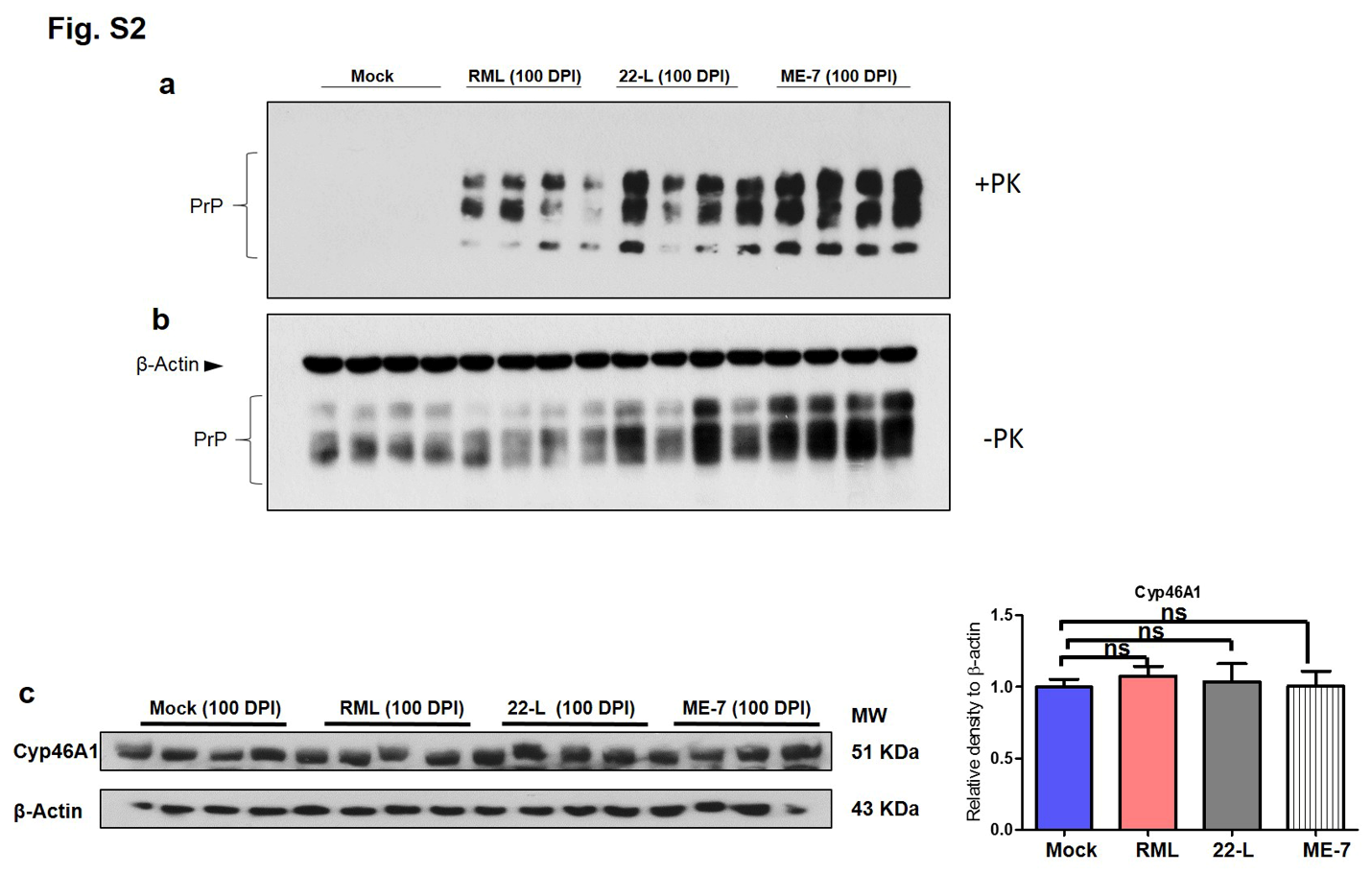
**

**Fig. S2.** **PrP and Cyp46A1 levels in mouse brain homogenates at 100 DPI.** **a, b** PrP levels at 100 DPI in brain homogenates of mice infected with different prion strains. PK-digested (PK+) and undigested (PK-) brain homogenates of mock and (RML, 22-L and ME-7) were used for immunoblotting and detection of PrP using mAb 4H11. No PrP^Sc^ was detected in mock group samples with +PK, while PrP^Sc^ was detected in all brain homogenates of prion infected mice at 100 DPI. β-actin was used as loading control. The PrP signal is high in all -PK samples at the expected ≈25–35 kDa range. In each case 4 different mice of mock and prion-infected (RML, 22-L and ME-7) groups were used. **c** The same brain homogenates of mock and prion-infected mice (RML, 22-L and ME-7) at 100 DPI were analyzed by immunoblot for Cyp46A1 levels. β-Actin was used as a loading control and was obtained after stripping of the same membrane. The histograms represent the means ± SEM (n = 4 mice/group) of three independent experiments. Significance = no significance (ns); ANOVA followed by Turkey’s post hoc test.


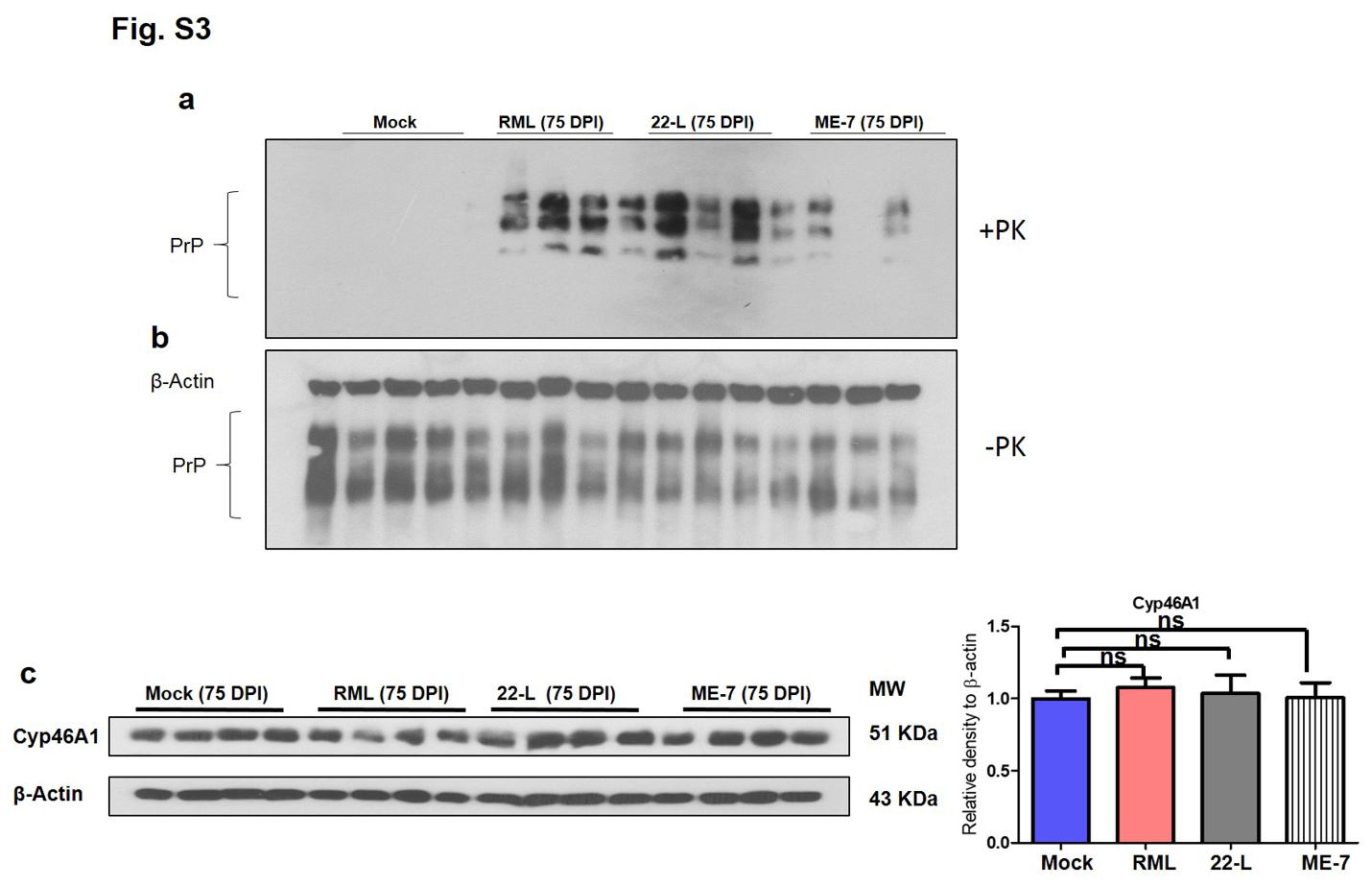


**Fig. S3. PrP and Cyp46A1 levels at early stage (75 DPI) of prion infection. a,b** PrP levels at 75 DPI in brain homogenates of mice infected different prion strains. The PK-digested (PK+) and undigested (PK-) brain homogenates of mock and prion-infected mice (RML, 22-L and ME-7) were used for immunoblotting and detection of PrP using mAb 4H11. No PrP^Sc^ was detected in mock group samples with +PK, while PrP^Sc^ was detected in all brain homogenates of prion infected mice at 75 DPI. β-actin was used as loading control. The PrP signal is high in all -PK samples at the expected ≈25–35 kDa range. In each case 4 different mice of mock and prion-infected (RML, 22-L and ME-7) groups were used. **c** The same brain homogenates of mock and prion-infected mice (RML, 22-L and ME-7) at 75 DPI were analyzed by immunoblot for Cyp46A1 levels. β-actin was used as a loading control and was obtained after stripping of the same membrane. The histograms represent the means ± SEM (n = 4 mice/group) of three independent experiments. Significance = no significance (ns); ANOVA followed by Turkey’s post hoc test.

**
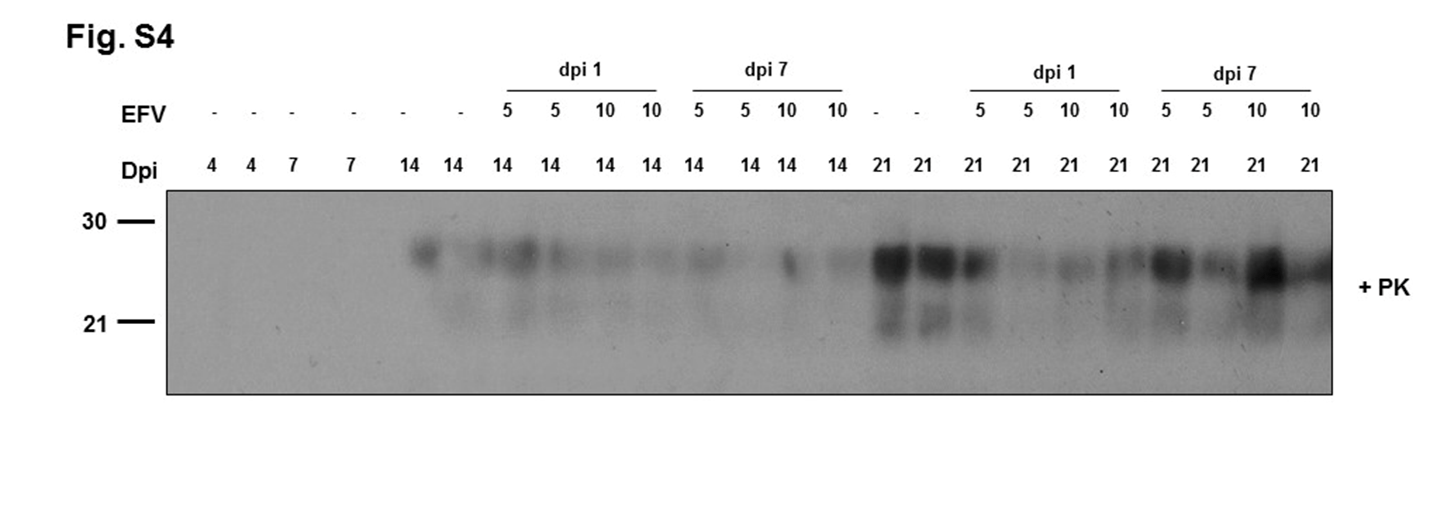
**

**Fig. S4: EFV treatment reduced PrP^Sc^ in the primary CGN culture.** PrP^Sc^ was detected in the lysates of RML-infected primary CGN cultures upon PK digestion by immunoblot using mAb

4H11. CGNs were treated every 4 days with EFV (5 µM, 10 µM) or vehicle until 21 days after RML infection and lysed at different time periods post infection as indicated.
